# Association of the angiopoietin-like protein 8 rs2278426 polymorphism and several environmental factors with serum lipid levels

**DOI:** 10.3892/mmr.2015.3825

**Published:** 2015-05-25

**Authors:** TAO GUO, RUI-XING YIN, JIAN WU, QUAN-ZHEN LIN, GUANG-YUAN SHI, SHAO-WEN SHEN, JIA-QI SUN, HUI LI, WEI-XIONG LIN, DE-ZHAI YANG

**Affiliations:** 1 Department of Cardiology, Institute of Cardiovascular Diseases, The First Affiliated Hospital, Guangxi Medical University, Nanning, Guangxi 530021, P.R. China; 2 Clinical Laboratory of the Affiliated Cancer Hospital, Guangxi Medical University, Nanning, Guangxi 530021, P.R. China; 3 Department of Molecular Biology, Medical Scientific Research Center, Guangxi Medical University, Nanning, Guangxi 530021, P.R. China

**Keywords:** lipids, apolipoproteins, angiopoietin-like protein 8, single nucleotide polymorphism, environmental factors

## Abstract

The present study was performed to examine the association of the angiopoietin-like protein 8 (*ANGPTL8*) rs2278426 single nucleotide polymorphism (SNP) and several environmental factors with serum lipid profiles in the Mulao and Han populations. A total of 879 individuals of the Mulao ethnic group and 865 individuals of the Han Chinese ethnic group were included. The serum apolipoprotein (Apo) B level was higher, however the serum ApoA1 level was lower in the Mulao individuals than in the Han individuals (P<0.05 and P<0.001, respectively). The genotypic and allelic frequencies, and the association with the *ANGPTL8* rs2278426 SNP were different between the Mulao and Han populations. The frequency of the A allele was 17.80% in Han individuals and 23.04% in Mulao individuals (P<0.001). The frequencies of GG, GA and AA genotypes were 68.79, 26.82 and 4.39% in the Han population, and 60.64, 32.65 and 6.71% in the Mulao population (P<0.005), respectively. A significant association between the SNP and serum lipid traits was only detected in Han females and not in Han males or in the Mulao population. The subjects with GA/AA genotypes had lower low-density lipoprotein cholesterol (LDL-C) and ApoB levels, and higher ApoA1 levels with a higher ApoA1/ApoB ratio than the subjects with the GG genotype in the Han population. Subgroup analyses revealed that the subjects with the GA/AA genotype had lower levels of total cholesterol, LDL-C and ApoB, and a higher ApoA1/ApoB ratio than the subjects with the GG genotype in Han females (P<0.05-P<0.001). Serum lipid parameters were also associated with several environmental factors, including dietary patterns, lifestyle, obesity, physical inactivity and hypertension, in the two ethnic groups (P<0.05-0.001). These findings suggest that there may be an ethnic- and gender-specific association of the rs2278426 SNP and serum lipid parameters.

## Introduction

It is well-established that mortality and morbidity occurring as a result of coronary artery disease (CAD) are a critical public health concern worldwide. Traditional CAD risk factors do not fully predict future CAD events (1,2) and adding modern biomarkers to the standard risk factors has, thus far, only proven to minimally improve individual risk prediction (3,4) thus underlining the requirement to identify novel biomarkers (5). Dyslipidemia, abnormal quantities of lipids in the blood, may be used as a biomarker indicating the susceptibility of an individual to CAD (6,7). Plasma total cholesterol (TC) (8), triglyceride (TG) (9), low-density lipoprotein cholesterol (LDL-C) (10), apolipoprotein (Apo) B (11), high-density lipoprotein cholesterol (HDL-C) (12), ApoA1 and the ApoA1/ApoB ratio (13) are traditionally monitored as predictors of dyslipidemia and also the main target for therapeutic intervention, by regulating blood lipid control (14). Dyslipidemia is well-recognized as a complex trait caused by multiple environmental and genetic factors (14,15), and their interactions (16,17). In twin and familial studies, 40–60% of the inter-individual variation in plasma lipid phenotypes was explained by heritable factors, such as single nucleotide polymorphisms (SNPs) (18–20).

Angiopoietin-like protein (ANGPTL) has a major role in trafficking and lipid metabolism. *ANGPTL*8, an ANGPTL family member, is located on chromosome 19 open reading frame 80 (c19orf80) in the corresponding intron of DOCK6. *ANGPTL*8 is expressed in the liver and adipose tissue, and circulates in the plasma of humans (21). A previous study (22) demonstrated that *ANGPTL*8 has an important role in lipoprotein metabolism through a functional interaction with *ANGPTL*3. Co-expression of *ANGPTL*8 with *ANGPTL*3 markedly increased plasma TG levels. Expression of *ANGPTL*8 was reduced by fasting in humans, while overexpression of *ANGPTL*8 resulted in hypertriglyceridemia (23). However, the variant in the *ANGPTL*8 gene has similar effects to complete *ANGPTL*3 deficiency, which is associated with low plasma levels of LDL-C, HDL-C and TG. Inhibition of *ANGPTL*8 may be able to provide a novel therapeutic strategy for reducing plasma lipoprotein levels (24). Recently, a common SNP adjacent to the *ANGPTL*8 locus, termed rs2278426, was identified as an SNP potentially affecting lipoprotein metabolism. The variant of the allele at this SNP was found to be associated with lower plasma LDL-C, HDL-C and TG levels resulting in a significantly reduced risk of CAD in humans (24). However, the effect of this SNP on serum lipid levels is not functionally validated and the mechanism for its action remains to be elucidated. Furthermore, the reproducibility of this association has not been detected in a Chinese population thus far.

China has been a multi-ethnic country since Ancient times (25). Among the 56 ethnic groups present in China, the Han ethnic group has the largest population. The Mulao ethnic group, also known as Mulam, is one of the 55 minorities with a population of 207,352 according to the fifth national census statistics of China in 2000. Of this population, 90% live in the Luocheng Mulao Autonomous County in the Guangxi Zhuang Autonomous Region. The history of this minority can be traced back to the Jin Dynasty (265–420 AD) (26). One previous study has demonstrated that the genetic association between Mulao ethnicity and other minorities in Guangxi was markedly closer than that between the Mulao and Han or Uighur ethnicity (27). In several previous studies, our group have revealed a significant association between several SNPs (28–31) and serum lipid levels in the Mulao population. To the best of our knowledge, the association between the rs2278426 SNP and serum lipid levels has not been previously examined in the Chinese population. Therefore, the aim of the present study was to assess the association of *ANGPTL8* rs2278426 SNP and several environmental factors with serum lipid phenotypes in the Mulao and Han populations.

## Materials and methods

### Study population

A total of 879 subjects of Mulao ethnicity who reside in Luocheng Mulao Autonomous County (Guangxi Zhuang Autonomous Region, China) comprising 407 males (46.30%) and 472 females (53.70%) and 865 participants of Han Chinese ethnicity residing in the same location, including 425 males (49.13%) and 440 females (50.87%) were randomly selected from our previous stratified randomized samples. The age range was between 15 and 80 years. The mean age of the Mulao participants was 52.69±14.99 years, whereas that of the Han subjects was 52.29±14.26 years. All participants were essentially healthy rural agricultural workers, with no evidence of disease associated with atherosclerosis, CAD or diabetes. Any participant who had a history of taking medication known to affect serum lipid levels (lipid-lowering drugs, including statins or fibrates, β-blockers, diuretics or hormones) was excluded from the study prior to the blood sample being obtained. The study design was approved by the Ethics Committee of the First Affiliated Hospital, Guangxi Medical University (Nanning, China). Informed consent was obtained from all participants.

### Epidemiological survey

The survey was conducted using internationally standardized methods, following a previously described protocol (32). Information on demographics, socioeconomic status and lifestyle factors was collected with standardized questionnaires. Alcohol consumption was quantified as the number of liangs (~50 g) of rice wine, corn wine, rum, beer or liquor consumed during the preceding 12 months. Alcohol consumption was categorized into groups of grams of alcohol consumed per day: 0 (nondrinker), ≤25 and >25. Cigarette smoking status was categorized into groups of cigarettes per day: 0 (non-smoker), ≤20 and >20. At the physical examination, several parameters, including height, weight and waist circumference were measured. Sitting blood pressure was measured three times with the use of a mercury sphygmomanometer (Jiangsu Yuyue Medical Equipment & Supply Co., Ltd., Danyang, China) after at least 5 min of rest and the average of the three measurements was used to indicate blood pressure. Systolic blood pressure was determined by the first Korotkoff sound and diastolic blood pressure was determined by the fifth Korotkoff sound. Body weight, to the nearest 50 g, was measured with a portable balance scale. Height was measured, to the nearest 0.5 cm, using a stadiometer (Shanghai Sangon Biological Engineering Technology & Services Co., Shanghai, China). From these two measurements, body mass index (BMI, kg/m^2^) was calculated. Waist circumference was measured with a non-stretchable measuring tape.

### Biochemical measurements

Venous blood samples of 5 ml were drawn after at least 12 h of fasting. A total of 2 ml of the sample was collected into a glass tube and used to determine serum lipid levels. The remaining 3 ml was transferred to tubes with anticoagulants (4.80 g/l citric acid, 14.70 g/l glucose and 13.20 g/l trisodium citrate; Shanghai Sangon Biological Engineering Technology & Services Co.) and used to extract DNA. Measurements of serum TC, TG, HDL-C and LDL-C levels in the samples were performed using enzymatic methods with a Ransod autoanalyzer (Randox Laboratories Ltd., Crumlin, UK and Daiichi Pure Chemicals Co., Ltd., Tokyo, Japan). Serum ApoA1 and ApoB levels were detected using the immunoturbidimetric immunoassay using a commercial kit (Randox Laboratories Ltd.). All determinations were performed with an autoanalyzer (Type 7170A; Hitachi Ltd., Tokyo, Japan) in the Clinical Science Experiment Center of the First Affiliated Hospital, Guangxi Medical University (27–30).

### DNA amplification and genotyping

Genomic DNA of the samples was isolated from peripheral blood leukocytes using the phenol-chloroform method (27–30). The extracted DNA was stored at 4°C until analysis. Genotyping of the *ANGPTL*8 rs2278426 SNP was determined by polymerase chain reaction and restriction fragment length polymorphism (PCR-RFLP). PCR amplification was performed using the following primer sequences: Forward 5′-CAGGAGTTCTATTGTGCGGC-3′ and reverse 5′-CCTGATGCAACTATCGCACC-3′ (Shanghai Sangon Biological Engineering Technology & Services Co.). Each 25 *µ*l PCR reaction mixture consisted of 2 *µ*l genomic DNA, 1 *µ*l each primer (10 pmol/l), 12.5 *µ*l of 2X Taq PCR Master mix (constituent: 20 mM Tris-HCl, pH 8.3, 100 mM KCl, 3 mM MgCl_2_, 0.1 U *Taq* Polymerase/*µ*l, 500 *µ*M dNTP each; Shanghai Sangon Biological Engineering Technology & Services Co.), and 8.5 *µ*l of ddH_2_O (DNase/RNase-free). PCR was performed with an initialization step of 95°C for 7 min, followed by 45 sec denaturing at 95°C, 45 sec of annealing at 60°C and 1 min of elongation at 72°C for 33 cycles. The amplification was completed with a final extension at 72°C for 7 min. Following electrophoresis on a 2.0% agarose gel with 0.5 *µ*g/ml ethidium bromide, the amplification products were visualized under ultraviolet light. Subsequently, each restriction enzyme reaction was performed with 5 *µ*l amplified DNA, 7.5 *µ*l nuclease-free water, 1 *µ*l of 10X buffer solution and 5 units *Bse*GI (*Bts*CI) (Shanghai Sangon Biological Engineering Technology & Services Co.) restriction enzyme in a total volume of 15 *µ*l, digested at 55°C overnight. Following restriction enzyme digestion of the amplified DNA, genotypes of the digestive products were identified using electrophoresis on 2% ethidium-bromide stained agarose gels and visualized under ultraviolet light. Genotypes were identified by an experienced technician blinded to the epidemiological and serum lipid results.

### DNA sequencing

The six samples (AA, AG and GG genotypes in the two ethnic groups) detected by the PCR-RFLP were also confirmed by direct sequencing. The PCR product was purified by low melting point gel electrophoresis and phenol extraction and then the DNA sequences were analyzed by Shanghai Sangon Biological Engineering Technology & Services Co.

### Diagnostic criteria

The normal values of serum TC, TG, HDL-C, LDL-C, ApoA1, ApoB levels and the ApoA1/ApoB ratio in our Clinical Science Experiment Center were 3.10–5.17, 0.56–1.70, 1.16–1.42, 2.70–3.10 mmol/l, 1.20–1.60, 0.80–1.05 g/l and 1.00–2.50, respectively. The individuals with TC>5.17 mmol/l and/or TG>1.70 mmol/l were defined as hyperlipidemic (33,34). Hypertension was diagnosed according to the criteria of the 1999 and 2003 World Health Organization-International Society of Hypertension Guidelines for the management of hypertension (35–37). The Cooperative Meta-analysis Group of China Obesity Task Force criteria were used for diagnosing an individual as overweight or obese (38). Normal weight, overweight and obese were defined as a BMI of <24, 24–28 or >28 kg/m^2^, respectively (39).

### Statistical analysis

Epidemiological data were recorded on a pre-designed form and managed with Microsoft Excel software (Microsoft, Redmond, WA, USA). All calculations were performed using SPSS software version 19.0 (IMB, Armonk, NY, USA) statistical package. The mean ± standard deviation (serum TG levels were presented as medians and interquartile ranges) and frequencies of baseline characteristics were calculated. Comparison of numerical variables, including age and body mass index, between the two groups was assessed using Student's unpaired t-test. Categorical variables were analyzed with a χ^2^ test or Fisher's exact test. Allele frequency was determined via direct counting and the standard goodness-of-fit test was used to test the Hardy-Weinberg equilibrium. Differences in genotype distribution between the groups were calculated using the χ^2^ test. The association of genotypes and serum lipid parameters were determined using analysis of covariance. Gender, age, BMI, blood pressure, alcohol consumption and cigarette smoking were adjusted for statistical analysis. Multivariate linear regression analysis with stepwise modeling was performed to evaluate the association of serum lipid levels with genotypes (GA/AA=1 and GG=2) and several environmental factors in the combined population and in, Mulao and Han groups, as well as males and females separately. P<0.05 was considered to indicate a statistically significant difference.

## Results

### General characteristics and serum lipid levels

The comparison of general characteristics and serum lipid levels between the Mulao and Han populations is summarized in Table I. Body weight, BMI, cigarette smoking levels and diastolic blood pressure were all lower in the Mulao population than in the Han population (P<0.05-0.001), whereas the levels of ApoB were higher in the Mulao individuals than in the Han individuals (P<0.001) and the level of ApoA1 was higher in the Han individuals than in the Mulao individuals (P<0.05). No significant differences were identified in the levels of systolic blood pressure, serum TC, TG, HDL-C, LDL-C, the ApoA1/ApoB ratio, age structure, gender ratio, height and the amount of alcohol consumption between the two ethnic groups (P>0.05 for all).

### Results of electrophoresis and genotyping

**Figure 1:**
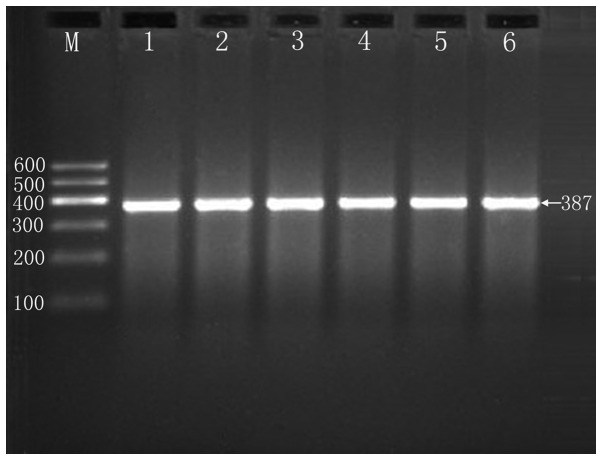
Electrophoresis of polymerase chain reaction products of the samples. Lane M is the 100 bp marker ladder; Lane 1–5 are samples, the 387 bp bands are the target genes.

**Figure 2:**
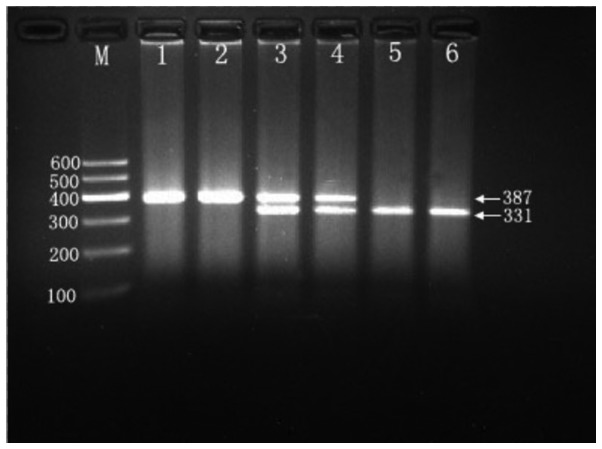
Genotyping of the angiopoietin-like protein 8 rs2278426 SNP. Lane M is the 100 bp marker Ladder; Lanes 1 and 2, GG genotype (387 bp); Lanes 3 and 4, AG genotype (387, 331 and 56 bp); and lanes 5 and 6, AA genotype (331 and 56 bp). The 56 bp segment is not visible in the gel owing to its fast migration speed.

After the genomic DNA of the samples was amplified using PCR and visualized with 2% agarose gel electrophoresis, products of 387 bp nucleotide sequences were observed in all samples (Fig. 1). The genotypes identified were termed according to the presence (A allele) or absence (G allele) of the enzyme restriction sites. Thus, the AA genotype is homozygous for the presence of the site (bands at 331 and 56 bp), the GA genotype is heterozygous for the presence and absence of the site (bands at 387, 331 and 56 bp) and the GG genotype is homozygous for the absence of the site (bands at 387 bp; Fig. 2). The 56 bp segment is not visible in the gel owing to its fast migratory speed. The genotypes of the rs2278426 SNP are in Hardy-Weinberg equilibrium.

### Results of sequencing

**Figure 3:**
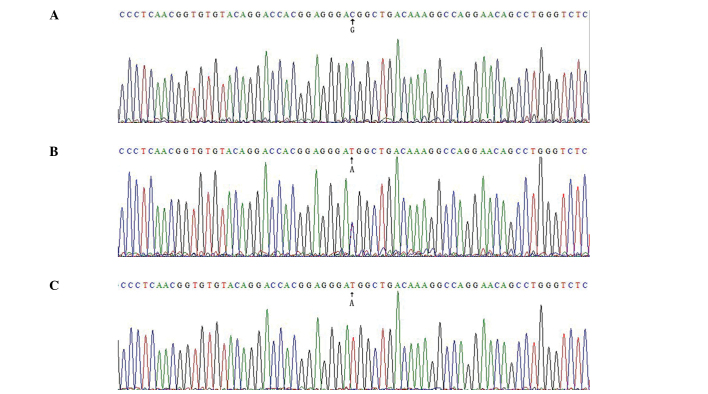
Nucleotide reverse sequence for part of angiopoietin-like protein 8 rs2278426. (A) GG genotype; (B) GA genotype; (C) AA genotype.

The results are presented as GG, GA and AA genotypes by PCR-RFLP, and the GG, GA and AA genotypes were also confirmed by reverse sequencing (Fig. 3), respectively. The reverse sequence used as the SNP site was upstream of the sequence, therefore the same base pairs in a row in front of the PCR products influence forward sequencing.

### Genotypic and allelic frequencies

The genotypic and allelic frequencies of the rs2278426 SNP in the *ANGPTL*8 gene are shown in Table II. The frequencies of G and A alleles were 82.20 and 17.80% in Han, and 76.96 and 23.04% in Mulao populations (P<0.001), respectively. The frequencies of GG, GA and AA genotypes were 68.79, 26.82 and 4.39% in the Han population and 60.64, 32.65 and 6.71% in the Mulao population (P<0.005), respectively. No differences in the genotypic frequencies between males and females in the two ethnic groups (P>0.05 for each) were identified.

### Genotypes and serum lipid levels

As shown in Tables III and IV, serum LDL-C, ApoA1 and ApoB levels and the ApoA1/ApoB ratio in the Han but not in the Mulao population were different among the genotypes (P<0.05-0.001). The subjects with the GA/AA genotypes had lower LDL-C and ApoB levels, and higher ApoA1 levels and a higher ApoA1/ApoB ratio than the subjects with the GG genotype. No differences were observed in serum lipid levels between the genotypes in the Mulao population (P>0.05). Subgroup analyses revealed that serum TC, LDL-C, ApoB levels and the ApoA1/ApoB ratio in Han females but not in males were different among the genotypes (P<0.05-0.001), the subjects with the GA/AA genotype had lower TC, LDL-C and ApoB levels, and a higher ApoA1/ApoB ratio than the subjects with the GG genotype (P<0.05-0.001). No differences in serum lipid levels were identified between the specific genotypes in Mulao females or males (P>0.05 for each).

### Relative factors for serum lipid parameters

The multiple linear regression analysis revealed that LDL-C, ApoA1, ApoB levels and the ApoA1/ApoB ratio in the Han population were correlated with the specific genotypes (P<0.05-0.001; Table V). As shown in Table VI, when serum lipid data were analyzed according to gender, TC, LDL-C and ApoB levels and the ApoA1/ApoB ratio in Han females were correlated with specific genotypes (P<0.05-0.001). Serum lipid parameters were also associated with age, gender, BMI, systolic and diastolic blood pressure, fasting blood glucose levels, cigarette smoking and alcohol consumption in the two ethnic groups (P<0.05-0.001, Tables V and VI).

## Discussion

In the present study, it was revealed that the serum ApoB level was higher but serum ApoA1 level was lower in the Mulao population than in the Han population. No significant differences in the levels of TC, HDL-C and LDL-C, and the ApoA1/ApoB ratio between the two ethnic groups were observed. These findings differ marginally from those of our previous studies (28–31). This difference may be associated with the sampling method. In addition, dyslipidemia is affected by environmental factors, including demographics, diet, alcohol consumption, cigarette smoking, obesity, exercise, hypertension (14,15) and genetic factors, including lipid-associated gene variants, and their interactions (16,17).

The Mulao ethnic group is a conservative and isolated minority. There are numerous cultural customs, including intra-ethnic marriage in this minority. The practice of arranged marriage is common, with decisions regarding future marriages often made during the individual's childhood. Divorce and remarriage are also allowed. Traditionally, females remain with their family until their first pregnancy. Prior to this, the females are often restricted from various social activities. The wife is usually four or five years older than the husband. Engagement and marriage are socially marked with a payment from the groom to the bride's family. Notably, the preferred marriage arrangement would be the groom's maternal cousin. Therefore, it is theorized that certain hereditary characteristics and genotypes of specific lipid metabolism-associated genes in this population may be different from those in the Han ethnic group (40–42).

To the best of our knowledge, the genotypic and allelic frequencies of the *ANGPTL*8 rs2278426 SNP have not been reported previously in different ethnic groups. In the present study, it was observed that the frequency of the A allele was lower in the Han population than in the Mulao population (17.80 vs. 23.04%; P<0.001). The distribution of the GG, GA and AA genotypes was also different between the two ethnic groups (P<0.005), the frequencies of GA and AA genotypes were lower in the Han than in the Mulao groups, respectively. No significant differences were identified in the genotypic and allelic frequencies between males and females in the two ethnic groups. These results suggest that the prevalence of the *ANGPTL*8 rs2278426 SNP may have an ethnic preponderance.

In the current study, it was also identified that serum LDL-C, ApoA1, ApoB levels and the ApoA1/ApoB ratio in the Han group were different among the genotypes (P<0.05-0.001). The subjects with the GA/AA genotype had lower LDL-C and ApoB levels and higher ApoA1 levels and a higher ApoA1/ApoB ratio than the subjects with the GG genotype, but these findings were restricted to females. Serum TC, LDL-C, ApoB levels and the ApoA1/ApoB ratio in Han females, but not in males were significantly different among the genotypes (P<0.05-0.001), the subjects with GA/AA genotype had lower TC, LDL-C, ApoB levels and a higher ApoA1/ApoB ratio than the subjects with the GG genotype. These findings suggest that there may be an ethnic- and gender-specific association of the *ANGPTL*8 rs2278426 SNP and serum lipid levels.

Environmental factors, including dietary patterns, lifestyle, obesity, physical inactivity and hypertension may substantially affect serum lipid levels (14,15). In the present study, it was also detected that serum lipid parameters were associated with age, gender, alcohol consumption, cigarette smoking, BMI, fasting blood glucose levels and blood pressure in the two ethnic groups. These data suggest that environmental factors also have an important role in determining serum lipid levels in the two populations. With the improvement in local living standards, the dietary intake of the Mulao population has changed gradually. However, the Mulao population generally eat cold foods and acidic and spicy dishes. Therefore soy beans and preserved vegetables are popular dietary sources. Animal offal is also commonly eaten, which has high levels of saturated fatty acids. High-fat diets, particularly those with abundant saturated fatty acids raise blood cholesterol concentrations and predispose individuals to CAD (43). Numerous studies have also stated that daily eating habits can markedly affect serum levels of ApoB, ApoA1 and their ratio, and which in turn can lead to an increased risk of CAD (44–46). In the present study, it was identified that the level of ApoB was higher in the Mulao than in the Han individual and the serum ApoA1 level was higher in the Han than in the Mulao individuals. This may be partly attributed to the difference in daily eating habits between the Mulao and Han populations.

The present study demonstrated that the genotypic and allelic frequencies of the *ANGPTL*8 rs2278426 SNP were different between the Mulao and Han populations. The subjects with the GA/AA genotype in the Han but not in the Mulao population had lower serum LDL-C and ApoB levels, a higher ApoA1 level and a higher ApoA1/ApoB ratio than the subjects with the GG genotype, but these results were restricted to females. Serum total TC, LDL-C, ApoB levels and the ApoA1/ApoB ratio in Han females were different among the genotypes. The subjects with the GA/AA genotype had lower TC, LDL-C, ApoB levels and a higher ApoA1/ApoB ratio than the subjects with the GG genotype. These results suggest that there may be an ethnic- and gender-specific association of the *ANGPTL*8 rs2278426 SNP and serum lipid levels in the populations investigated in the present study.

In conclusion, the present study detected an association of the *ANGPTL*8 rs2278426 SNP and numerous environmental factors with serum lipid profiles; however, there are still many environmental and genetic factors, and their interactions, that have yet to be measured. Therefore, the association of gene-gene, gene-environment, and environment-environment interactions with serum lipid levels remains to be elucidated.

## Figures and Tables

**Table I:** Comparison of demographic, lifestyle characteristics and serum lipid levels between the Han and Mulao populations.

Parameter	Han	Mulao	t (χ^2^)	P-value
No. of patients	865	879		
Male/female	425/440	407/472	1.400	0.273
Age (years)	52.29±14.26	52.69±14.99	−0.581	0.561
Height (cm)	155.00±7.96	155.37±8.13	−0.951	0.342
Weight (kg)	54.17±9.25	52.99±9.34	2.661	0.008
Body mass index (kg/m^2^)	22.51±3.33	21.89±3.09	4.030	0.000
Waist circumference	74.74±7.94	75.27±8.54	1.193	0.233
Smoking status (n %)			8.208	0.017
0 g/day (non-smoker)	592 (68.44)	653 (74.29)		
≤20 cigarettes/day	108 (12.49)	80 (9.10)		
>20 cigarettes/day	165 (19.08)	146 (16.61)		
Alcohol consumption, n (%)			3.708	0.157
0 g/day (non-drinker)	650 (75.14)	658 (74.86)		
≤25 g/day	3 (0.35)	10 (1.14)		
>25 g/day	212 (24.51)	211 (24.00)		
Systolic blood pressure (mmHg)	130.74±18.52	129.78±21. 86	0.989	0.323
Diastolic blood pressure (mmHg)	82.71±10.70	81.27±11.60	2.695	0.007
Pulse pressure (mmHg)	48.03±14.23	48.51±16.40	−0.654	0.513
Glucose (mmol/l)	6.19±1.77	6.03±1.70	1.851	0.064
Total cholesterol (mmol/l)	5.06±0.95	5.02±1.11	0.669	0.504
Triglyceride (mmol/l)	1.08 (0.80)	1.06 (0.79)	−1.153	0.249
HDL-C (mmol/l)	1.75±0.56	1.75±0.45	0.215	0.830
LDL-C (mmol/l)	2.92±0.82	2.97±0.87	−1.175	0.240
Apo A1 (g/l)	1.36±0.26	1.33±0.40	1.990	0.047
ApoB (g/l)	0.87±0.20	0.98±0.57	−5.898	0.000
ApoA1/ApoB	1.65±0.50	1.60±0.95	1.237	0.216

Values of triglycerides were expressed as the median (interquartile range). The difference between the two ethnic groups was determined using the Wilcoxon-Mann-Whitney test. HDL-C, high-density lipoprotein cholesterol; LDL-C, low-density lipoprotein cholesterol; Apo, Apolipoprotein.

**Table II:** Comparison of the genotype and allelic frequencies of angiopoietin-like protein 8 rs2278426 SNP in the Han and Mulao populations.

Group	n	Genotype, n (%)					Allele, n (%)			
		GG	GA	AA	χ^2^	P-value	G	A	χ^2^	P-value
Han +	865	595 (68.79)	232 (26.82)	38 (4.39)	13.671	0.001	1422 (82.20)	308 (17.80)	14.688	0.000
Mulao	879	533 (60.64)	287 (32.65)	59 (6.71)			1353 (76.96)	405 (23.04)		
Han										
Male	425	288 (67.76)	120 (28.24)	17 (4.00)	1.044	0.593	696 (81.88)	154 (18.12)	0.113	0.737
Female	440	307 (69.77)	112 (25.46)	21 (4.77)			726 (82.50)	154 (17.50)		
Mulao										
Male	407	243 (59.70)	129 (31.70)	35 (8.60)	4.343	0.114	615 (75.55)	199 (24.45)	1.699	0.192
Female	472	290 (61.44)	158 (33.48)	24 (5.08)			738 (78.18)	206 (21.82)		

**Table III:** Comparison of the genotypes and serum lipid levels in the Han and Mulao populations.

Genotype	n	TC (mmol/l)	TG (mmol/l)	HDL-C (mmol/l)	LDL-C (mmol/l)	ApoA1 (g/l)	ApoB (g//l)	ApoA1/ApoB
Han								
GG	595	5.08±0.96	1.09(0.80)	1.74±0.61	2.96±0.81	1.34±0.26	0.88±0.19	1.61±0.47
GA/AA	270	4.99±0.94	1.02(0.78)	1.77±0.42	2.84±0.86	1.39±0.25	0.85±0.21	1.74±0.55
F-value		3.397	0.040	3.051	5.816	4.268	8.186	12.161
P-value		0.065	0.841	0.081	0.016	0.039	0.004	0.000
Mulao								
GG	533	5.04±1.11	1.04(0.76)	1.76±0.47	2.97±0.87	1.33±0.40	0.97±0.56	1.62±1.02
GA/AA	346	5.00±1.12	1.09(0.84)	1.73±0.41	2.96±0.88	1.33±0.39	1.00±0.58	1.58±0.84
F-value		−0.190	−1.344	−1.312	−0.048	−0.571	−0.647	−0.857
P-value		0.849	0.179	0.189	0.962	0.568	0.518	0.392

Value of TGs were expressed as the (interquartile range). The difference between the genotypes was determined using the Wilcoxon-Mann-Whitney test. TC, total cholesterol; TG, triglyceride; HDL-C, high-density lipoprotein cholesterol; LDL-C, low-density lipoprotein cholesterol; ApoA1, Apolipoprotein A1; ApoB, Apolipoprotein B; ApoA1/ApoB, the ratio of Apolipoprotein A1 to Apolipoprotein B.

**Table IV:** Comparison of the genotypes and serum lipid levels between males and females in the Han and Mulao populations.

Ethnicity/genotype	n	TC (mmol/l	TG (mmol/l	HDL-C (mmol/l	LDL-C (mmol/l	ApoA1 (g/l)	ApoB (g/l)	ApoA1/ApoB
Han/male								
GG	288	4.96±0.98	1.07(0.77)	1.71±0.43	2.85±0.80	1.35±0.26	0.84±0.16	1.66±0.43
GA/AA	137	4.95±0.86	1.07(0.71)	1.76±0.42	2.75±0.89	1.38±0.24	0.83±0.19	1.76±0.55
F-value		−0.070	−0.229	−1.271	−1.280	−1.343	−0.871	−1.451
P-value		0.944	0.819	0.204	0.201	0.179	0.384	0.147
Han/female								
GG	307	5.20±0.92	1.09(0.83)	1.78±0.74	3.06±0.80	1.34±0.25	0.91±0.21	1.56±0.50
GA/AA	133	5.04±1.01	0.98(0.83)	1.79±0.42	2.93±0.82	1.39±0.27	0.86±0.22	1.73±0.55
F-value		−2.464	−0.594	−1.256	−2.197	−1.561	−3.216	−3.578
P-value		0.014	0.553	0.209	0.028	0.119	0.001	0.000
Mulao/male								
GG	243	5.07±1.01	1.08(0.77)	1.76±0.53	2.94±0.82	1.35±0.42	1.03±0.62	1.53±0.676
GA/AA	164	5.03±1.17	1.16(0.86)	1.70±0.42	2.95±0.83	1.32±0.40	1.04±0.63	1.50±0.69
F-value		−0.115	−1.131	1.484	−0.144	1.375	−0.015	−1.098
P-value		0.908	0.258	−0.138	0.886	−0.169	0.988	0.272
Mulao/female								
GG	290	5.01±1.18	1.01(0.76)	1.76±0.42	2.99±0.91	1.31±0.39	0.93±0.50	1.68±1.24
GA/AA	182	5.00±1.08	1.04(0.82)	1.76±0.40	2.97±0.91	1.34±0.37	0.96±0.53	1.66±0.94
F-value		-0.185	−0.744	−0.301	−0.091	−0.645	−0.846	−0.190
P-value		0.853	0.457	0.763	0.928	0.519	0.398	0.849

Value of TGs were expressed as the median (interquartile range). The difference between the genotypes was determined by the Wilcoxon-Mann-Whitney test. TC, total cholesterol; TG, triglyceride; HDL-C, high-density lipoprotein cholesterol; LDL-C, low-density lipo-protein cholesterol; ApoA1, Apolipoprotein A1; ApoB, Apolipoprotein B; ApoA1/ApoB, the ratio of Apolipoprotein A1 to Apolipoprotein B.

**Table V:** Association between serum lipid parameters and relative factors in the Han and Mulao populations.

A, Han and Mulao						

Lipid parameter	Risk factor	B	Std. error	β	t	P-value
TC	Age	0.010	0.002	0.136	5.520	0.000
	Alcohol consumption	0.070	0.029	0.058	2.446	0.015
	Diastolic blood pressure	0.008	0.002	0.085	3.453	0.001
	Body mass index	0.043	0.008	0.135	5.587	0.000
	Glucose	0.021	0.014	0.035	1.466	0.143
TG	Waist circumference	0.048	0.004	0.304	13.099	0.000
	Cigarette smoking	0.218	0.038	0.129	5.770	0.000
	Diastolic blood pressure	0.008	0.003	0.070	2.982	0.003
	Ethnic group	−0.155	0.059	−0.059	−2.643	0.008
	Glucose	0.053	0.017	0.070	3.023	0.003
	Age	−0.004	0.002	−0.044	−1.891	0.059
HDL-C	Waist circumference	−0.008	0.002	−0.1260	−3.787	0.000
	Alcohol consumption	0.094	0.017	0.158	5.572	0.000
	Gender	0.120	0.029	0.119	4.132	0.000
	Age	0.002	0.001	0.047	2.007	0.045
	Body mass index	−0.018	0.005	−0.113	−3.460	0.001
LDL-C	Age	0.009	0.001	0.147	6.135	0.000
	Body mass index	0.044	0.006	0.166	7.053	0.000
	Ethnic group	0.077	0.040	0.045	1.920	0.055
	Glucose	0.026	0.012	0.053	2.220	0.027
ApoA1	Alcohol consumption	0.103	0.011	0.265	9.287	0.000
	Body mass index	−0.010	0.002	−0.096	−4.062	0.000
	Gender	0.068	0.019	0.101	3.564	0.000
ApoB	Waist circumference	0.010	0.001	0.196	8.411	0.000
	Ethnic group	0.126	0.020	0.147	6.337	0.000
	Glucose	0.021	0.006	0.086	3.682	0.000
ApoA1/ApoB	Waist circumference	−0.011	0.003	−0.114	−3.394	0.001
	Glucose	−0.021	0.011	−0.048	−1.973	0.049
	Body mass index	−0.026	0.008	−0.110	−3.323	0.001
	Alcohol consumption	0.105	0.025	0.118	4.146	0.000
	Gender	0.172	0.044	0.113	3.920	0.000
	Ethnic group	−0.071	0.036	−0.046	−1.975	0.048
	Age	−0.004	0.001	−0.068	−2.837	0.005

B, Han						

Lipid parameter	Risk factor	B	Std. error	β	t	P-value
TC	Waist circumference	0.016	0.004	0.130	3.759	0.000
	Age	0.009	0.002	0.137	3.876	0.000
	Alcohol consumption	0.104	0.037	0.094	2.777	0.006
	Diastolic blood pressure	0.013	0.003	0.151	4.321	0.000
	Glucose	0.024	0.018	0.045	1.324	0.186
TG	Waist circumference	0.061	0.006	0.314	9.739	0.000
	Cigarette smoking	0.393	0.061	0.201	6.460	0.000
	Glucose	0.114	0.029	0.130	4.006	0.000
	Diastolic blood pressure	0.011	0.005	0.076	2.302	0.022
	Age	−0.07	0.004	−0.062	−1.876	0.061
HDL-C	Waist circumference	−0.013	0.002	−0.186	−5.348	0.000
	Gender	0.138	0.045	0.123	3.082	0.002
	Alcohol consumption	0.080	0.026	0.123	3.078	0.002
LDL-C	Age	0.010	0.002	0.172	5.010	0.000
	Body mass index	0.041	0.008	0.164	4.962	0.000
	Glucose	0.035	0.016	0.074	2.157	0.031
	Genotype	0.019	0.048	0.013	11.857	0.000
ApoA1	Alcohol consumption	0.091	0.012	0.304	7.685	0.000
	Body mass index	−0.017	0.003	−0.225	−6.812	0.000
	Gender	−0.014	0.007	−0.028	−2.109	0.035
	Cigarette smoking	0.047	0.013	0.145	3.536	0.000
	Genotype	0.077	0.022	0.150	3.476	0.001
ApoB	Waist circumference	0.006	0.001	0.230	5.374	0.000
	Glucose	0.015	0.003	0.136	4.363	0.000
	Alcohol consumption	0.022	0.007	0.097	3.028	0.003
	Systolic blood pressure	0.001	0.000	0.136	4.311	0.000
	Body mass index	0.007	0.002	0.124	2.912	0.004
	Genotype	0.001	0.011	0.004	1.025	0.035
ApoA1/ApoB	Waist circumference	−0.010	0.003	−0.162	−3.658	0.000
	Systolic blood pressure	−0.002	0.001	−0.084	−2.552	0.011
	Glucose	−0.014	0.009	−0.049	−1.506	0.132
	Body mass index	−0.031	0.007	−0.205	−4.653	0.000
	Cigarette smoking	0.063	0.020	0.100	3.082	0.002
	Gender	0.126	0.041	0.126	3.068	0.002
	Genotype	−0.030	0.011	−0.027	−2.731	0.006

C, Mulao						

Lipid parameter	Risk factor	B	Std. error	β	t	P-value
TC	Age	0.010	0.002	0.138	4.163	0.000
	Body mass index	0.053	0.012	0.148	4.465	0.000
TG	Waist circumference	0.037	0.004	0.309	9.603	0.000
	Alcohol consumption	0.114	0.038	0.096	2.996	0.003
HDL-C	Waist circumference	−0.007	0.002	−0.129	−2.738	0.006
	Alcohol consumption	0.065	0.017	0.123	3.747	0.000
	Body mass index	−0.023	0.007	−0.159	−3.423	0.001
	Age	0.002	0.001	0.052	1.586	0.113
LDL-C	Age	0.007	0.002	0.128	3.877	0.000
	Body mass index	0.048	0.009	0.170	5.160	0.000
ApoA1/ApoB	Alcohol consumption	0.079	0.015	0.171	5.142	0.000

TC, total cholesterol; TG, triglyceride; HDL-C, high-density lipoprotein cholesterol; LDL-C, low-density lipoprotein cholesterol; ApoA1, Apolipoprotein A1; ApoB, Apolipoprotein B; ApoA1/ApoB, the ratio of Apolipoprotein A1 to Apolipoprotein B; Std. error, standard error.

**Table VI:** Association between serum lipid parameters and relative factors in the males and females of the Han and Mulao populations.

A, Han/male						

Lipid parameter	Risk factor	B	Std. error	β	t	P-value
TC	Diastolic blood pressure	0.024	0.004	0.284	6.099	0.000
	Alcohol consumption	0.098	0.042	0.109	2.324	0.021
	Glucose	0.029	0.023	0.059	1.259	0.209
TG	Waist circumference	0.080	0.010	0.355	7.793	0.000
	Cigarette smoking	0.487	0.093	0.234	5.250	0.000
	Diastolic blood pressure	0.002	0.008	0.012	0.272	0.785
	Glucose	0.080	0.045	0.079	−1.769	0.078
HDL-C	Body mass index	−0.035	0.006	−0.299	−6.302	0.000
	Alcohol consumption	0.081	0.020	0.190	4.016	0.000
LDL-C	Cigarette smoking	−0.181	0.043	−0.196	−4.153	0.000
	Body mass index	0.034	0.010	0.151	3.207	0.001
ApoA1	Alcohol consumption	0.094	0.013	0.336	7.236	0.000
	Body mass index	−0.019	0.004	−0.247	−5.439	0.000
	Cigarette smoking	0.048	0.014	0.0151	3.343	0.001
ApoB	Body mass index	0.008	0.003	0.153	2.716	0.007
	Glucose	0.017	0.005	0.160	3.641	0.000
	Alcohol consumption	0.019	0.009	0.099	2.217	0.027
	Diastolic blood pressure	0.004	0.001	0.209	4.636	0.000
	Waist circumference	0.004	0.001	0.179	3.188	0.002
ApoA1/ApoB	Body mass index	−0.053	0.006	−0.386	−8.525	0.000
	Cigarette smoking	0.086	0.026	0.151	3.367	0.001
	Alcohol consumption	0.088	0.023	0.176	3.820	0.000

B, Han/female						

Lipid parameter	Risk factor	B	Std. error	β	t	P-value
TC	Alcohol consumption	−0.113	0.170	−0.030	−0.665	0.506
	Age	0.024	0.003	0.320	7.017	0.000
	Body mass index	0.058	0.015	0.172	3.781	0.000
	Genotype	0.072	0.036	0.035	1.982	0.048
TG	Waist circumference	0.045	0.007	0.278	6.135	0.000
	Diastolic blood pressure	0.017	0.005	0.147	3.262	0.001
	Glucose	0.122	0.030	0.177	4.019	0.000
HDL-C	Waist circumference	−0.008	0.004	−0.093	1.951	0.052
LDL-C	Body mass index	0.049	0.013	0.170	3.753	0.000
	Age	0.021	0.003	0.324	7.109	0.000
	Alcohol consumption	−0.218	0.144	−0.069	−1.510	0.132
	Genotype	−0.065	0.031	−0.037	−2.075	0.039
ApoA1	Cigarette smoking	0.082	0.051	0.076	1.598	0.111
	Diastolic blood pressure	−0.003	0.001	−0.125	−2.640	0.009
ApoB	Body mass index	0.019	0.003	0.296	6.758	0.000
	Glucose	0.015	0.005	0.133	2.942	0.003
	Age	0.003	0.001	0.240	5.153	0.000
	Cigarette smoking	−0.097	0.039	−0.112	−2.475	0.014
	Genotype	0.003	0.008	0.007	0.434	0.000
ApoA1/ApoB	Body mass index	−0.046	0.008	−0.274	−6.037	0.000
	Cigarette smoking	0.314	0.106	0.136	2.953	0.003
	Systolic blood pressure	−0.002	0.001	−0.074	−1.477	0.140
	Age	−0.007	0.002	−0.191	−3.717	0.000
	Genotype	−0.019	0.017	−0.017	−1.139	0.000

C, Mulao/male						

Lipid parameter	Risk factor	B	Std. error	β	t	P-value
TC	Body mass index	0.060	0.018	0.169	3.422	0.001
TG	Waist circumference	0.049	0.006	0.359	7.751	0.000
HDL-C	Alcohol consumption	0.100	0.023	0.203	4.253	0.000
	Waist circumference	−0.013	0.003	−0.234	−4.906	0.000
	Age	0.001	0.002	0.036	0.749	0.455
LDL-C	Body mass index	0.043	0.013	0.156	3.182	0.002
ApoA1	Alcohol consumption	0.116	0.020	0.280	5.866	0.000
ApoB	Waist circumference	0.009	0.003	0.127	2.580	0.010
ApoA1/ApoB	Alcohol consumption	0.130	0.033	0.190	3.967	0.000
	Waist circumference	−0.016	0.004	−0.208	−4.336	0.000

D, Mulao/female						

Lipid parameter	Risk factor	B	Std. error	β	t	P-value
TC	Age	0.015	0.003	0.204	4.513	0.000
TG	Body mass index	0.054	0.010	0.253	5.653	0.000
HDL-C	Body mass index	−0.031	0.006	−0.234	−5.206	0.000
LDL-C	Body mass index	0.051	0.013	0.177	3.962	0.000
	Age	0.012	0.003	0.196	4.381	0.000
ApoB	Glucose	0.047	0.016	0.133	2.940	0.003
	Waist circumference	0.012	0.003	0.192	4.252	0.000
	Cigarette smoking	0.392	0.245	0.072	1.599	0.011
ApoA1/ApoB	Cigarette smoking	−0.602	0.503	−0.055	−1.197	0.232
	Waist circumference	−0.016	0.007	−0.111	−2.431	0.015
	Age	−0.010	0.003	−0.128	−2.793	0.005

TC, total cholesterol; TG, triglyceride; HDL-C, high-density lipoprotein cholesterol; LDL-C, low-density lipoprotein cholesterol; ApoA1, Apolipoprotein A1; ApoB, Apolipoprotein B; ApoA1/ApoB, the ratio of Apolipoprotein A1 to Apolipoprotein B; Std. error, standard error.
